# The negative effects of new screens on the cognitive functions of young children require new recommendations

**DOI:** 10.1186/s13052-021-01174-6

**Published:** 2021-11-06

**Authors:** Osika Eric

**Affiliations:** Department of Pediatrics, Saint Camille Hospital, 2 Rue des Pères Camilliens, 94360 Bry-sur-Marne, France

**Keywords:** Children, Infant, Television, Smartphone, E-book, Screen, Cognitive function, Academic outcomes, Language, Technoference

## Abstract

Television studies have shown that some negative effects of screens could depend on exposure time, but more importantly on the characteristics of the child, the type of content viewed, and the context in which it is viewed. Studies on newer screens show that these factors are still valid but new ones now play a negative role: portable screens increase the duration of exposure and lowered the age at which exposure begins. More worryingly, new screen persuasive designs and dark patterns largely used incite more frequent use, attracting the attention of children and parents, resultantly interfering deeply in parent/child relationships. In this text we suggest that current academic recommendations have to be more broadly shared but also that new recommendations are needed: especially to advise parents not to let their screen interactions compete with real interactions with their child which are the core of learnings (especially language) and emotional regulations but also of their security.

In recent decades, the age at which a child is first exposed to television has dramatically lowered. In a recent study in Singapore, 28% of children under the age of 6 months experienced a daily exposure [1]. In 2020, the last US national survey of 1400 parents conducted by the independent association Common Sense Media (CSM), found that American children aged under 2 spent daily 45 min in front of television. In parallel, the overall time 0–8 years-old children spend on mobile devices has been constantly increasing over the past decade: 5 min per day in 2011, 15 min in 2013 and 55 min in the last CSM survey [2]. Nowadays new screens are present anytime anywhere: online videos can be accessed any time of the day, tablets and smartphones permit to view them in the car, in the restaurant even in the pediatrician waiting room. Research on the effects of television has been undertaken for several decades, but studies on the effects of this new multi-screen environment have only been published recently. How can one assess the effects of these new media consumption? A first step towards better understanding could be provided by previous studies on “traditional” media, like television and video but specific issues related to these new screens are likely to come to light.

## The known effects of traditional media (TV and video)

The effects of television on cognitive functions have been assessed in many longitudinal and cross-sectional studies for over 20 years, and on varying age groups [3]. Regardless of the specific context or content of screen viewing, most of studies have shown that time spent in front of a television is correlated with a decrease in cognitive performance, when considering language, concentration, or more general developmental features [4, 5].

### Television in background modifies family’s interactions

Studies have shown a significant decrease in human interaction when TV is turned on at home, even if a child is not directly engaged with it. This effect has been widely studied in the past twenty years as it majorly impacts toddlers. When television is turned on in the background, children play for a shorter period of time and their attention is disrupted for short (less than 2 s), but repeated, periods [6]. When television is turned on, it interferes with parent-child interactions. Parents’ responses to their child’s solicitations, such as participating in the child’s game, are shown to be reduced [7]. Verbal communication is altered quality-wise (with a decrease in number of new words) as well as quantity-wise (with up to a 1000 word decrease in total words exchanged between parents and children in relation to per hour of television exposure) [8]. These studies are the first to highlight an essential notion: screens act as a “barrier” between parents and children, deeply modifying family interactions at a time of life when they are the very basis for all learning.

### Emotional disorder and TV: a bidirectional correlation

As early as 2006, a study showed that children who watched television before the age of 4 protested more than others when the television was turned off at age 6. In 2010, a Japanese study of 479 children linked high levels of television exposure time at 18 months with attention and behavioral disorders at 30 months [9]. Other teams have since confirmed these correlations [10]. These large cross-sectional studies also revealed that children with character disorders were exposed to screens more often than any others. The studies revealed that parents were more likely to put difficult children in front of screens to calm them, or to occupy them in order to make time for themselves [11]. Thus, paradoxically, whilst television is often used by parents to manage their child’s difficult behavior, such actions have adverse long-term effects [12]..

In 2017, Wu and his team, in a Chinese study on 8900 children aged 3 to 6 years, showed the existence of an increased risk of total difficulties, emotional symptoms, conduct problems, hyperactivity, peer problems, and prosocial problems, as well as behavioral symptoms mimicking autism spectrum disorder, in children exposed to more than 2 h of television per day [13]. Moreover, Numata-Uematsu reported a 5-year old boy who, having been exposed to media during his early development, had later displayed neurobehavioural symptoms that mimicked autism [14]. Similar observations have been reported and discussed by clinicians worldwide [15–17].

### A massive effect of TV on children’s sleep

This is a major factor with considerable repercussions. Screens have an impact in the youngest and the oldest children [18, 19], on sleep both quantitatively (bedtime, sleep time, and total sleep time) and qualitatively (restful sleep or not) [1, 20, 21]. The effect is particularly pronounced if the television is watched just before bedtime or if it is in the child’s bedroom [22].

## The effects of new media are not so different from theses described with television

The category of new media includes new video-on-demand platforms; either free ones (e.g., YouTube) or those for which subscription is required (e.g., Netflix). In most of the cases a version for children is available. Mobile screens include so-called interactive tablets, e-books, and mobile phones which are ever-increasingly connected to the internet.

### Cross-sectional “all-screen” studies show similar effects

These studies cover all categories of screens currently available (television, video, tablets and phones) and their findings are analogous with previous studies on television alone. They found a similar negative correlation between screen exposure and cognitive, academic and executive skills [5, 23]. This is likely due to that fact that new screens are essentially used for entertainment purposes, in front of which the child watches videos or a television program repeatedly, in the same manner as when in front of a traditional television: alone, in front of exclusively recreational content [2, 24, 25]. A significant study was published in 2018, which portrayed a temporal relationship of correlation between screen time and cognitive indexes (Age Stage Questionnaire or ASQ), thus demonstrating a causal relationship [26]. In that study, Madigan followed 2441 Canadian children from the ages of 24 to 60 months and compared “all-screen” time experienced by the children, and a cognitive development index (ASQ3) assessed between 12 and 24 months later. Madigan’s results revealed a negative correlation between screen time at 24 months and cognitive tests 12 months later, and a negative correlation between screen time at 36 months and cognitive tests 24 months later. In the cases in which digital screen time was high at the first assessment, the cognitive test was degraded a few months later when the reverse correlation was not found. This one-sided temporal relationship favors a causal effect between screen time and cognitive deficit [26]. In 2019, Mac Neil published a study of 185 Australian children between the ages of 3 and 5. An excessive consumption of “all-screens” was significantly associated with an increase in difficult behaviors and general difficulties 12 months later. Children who spent more than 30 min per day on a mobile application exhibited, 12 months later, a decrease in one of the main components of their executive skills: their inhibition score [27].

### The heavy use of mobile screens could be a source of language delay

In 2019, a Canadian study conducted on a neonatal cohort of 893 children showed a correlation between handheld device use (iPhones, iPads, Tablets, Nintendo DS) and language delay at 18 months. At 18 months, 22% of children in the cohort used a mobile screen for an average of 15.7 min per day. The greater the time spent on a mobile phone, the greater the risk of late language skills: for every thirty minutes of additional screen time, on average, the authors reported a 49% increase in the risk of late language skills [28]. Moon, a South Korean author, studied 117 children aged 3 to 5 and their mobile phone use; he noted a similar correlation between mobile screen time and late language skills in 3 year old children [29].

### Mobile screens and emotional disorder: the same bidirectional effect

Hosokawa studied 1642 6 year old Japanese children exclusively on the effects of mobile phone use: those who used mobiles for over 60 min were found to have more behavioral and concentration problems than non-users [30]. Recent studies on the use of mobile screens have shown that they are most often used to calm difficult children [24, 31]. Poulain and Cliff found bi-directional correlations between moving screens and character disturbances, just as it had previously been demonstrated in regard to television [32]. In 2018, Cliff observed 2300 Australian children, all 2, 4 or 6 years old. Children who had the lowest “all screen” time at the age of 2 had the highest behavior score at the age of 4, and, correspondingly, children with the worst behavior score at the age of 4 had the highest screen time at the age of 6 [33].

### Mobile screens are much more deleterious for children’s sleep

Several recent studies have shown that child’s sleep is particularly impacted by the use of new digital devices [1, 34–36]. An English study of 715 children aged 6 to 36 months found a significant association between frequent daily device use and sleep, noting a specific decrease in total sleep time and a delay in falling asleep. Each additional hour of mobile phone use during the day was found to reduce night-time sleep by 26 min and increase nap-time sleep by 10 [37]. This could have been due to mobile phones simply being more available for use, but also due to the blue light emitted by new LED screens which inhibits melatonin and causes a shift in its secretion [38].

### Mobile screens are wider and sooner used

It is evident from daily medical practice that the specific “handheld” nature of such devices enhances the presence of screens in family. Some parents state that they systematically use their mobiles whilst feeding their children, to the extent that some children are unable to eat without the presence of a mobile phone. Other toddlers fall asleep every night with a well-known Interactive Pad near close to them. This new accessibility has not yet been assessed or taken into account in studies. Nonetheless, this feature clearly exacerbates the risk of overexposure to children, not only in terms of duration but also precocity. An infant, almost from birth (many fathers film their child’s birth with a mobile phone), will be witness to the constant presence of a mobile phone in a parent’s hand, and will quickly understand the unavoidable nature of this device. This “joint attention» created by the parent with the digital device, at a time in which human interactive skills are essential, is not, probably, without consequences [39].

### The paradox of E-books and digital books’ use

Studies on digital books are increasingly frequent and the most recent ones have found notably contrasting results [40]. Despite the peculiarity of its content, the digital book is like any other interactive screen. Its use must adhere to specific rules in order for it to prove beneficial, and not disrupt parent-child interactions [41]. In a recent study authors noticed that when reading an animated e-book, exchanges between parents and children were lessened in quantity as well as quality (compared to the reading of a traditional book), despite the fact that the shared experience between a parent and child over a book is presumed to be rewarding [42]. Once again, as in the case of background television, the screen becomes a barrier between the parent and child.

### Mobile screens interfere in family relationships

In 2015 a laboratory study showed that parents who used their mobile phones during meal-times had less verbal and non-verbal interaction with their children. This author had initially noticed this effect during an authentic family meal and found their suspicions regarding consequential negative interactions, and bad behavior of the children, to be confirmed by their study [43, 44]. Similar results were obtained while parents were “watching” their child in a playground or at the swimming-pool [45, 46]. In a survey completed through online questionnaires, 10% of parents reported that their child got hurt while they were busy using social networking platforms on their mobile phones [47]. In a small study of 38 dyads of 2 year old children and their mothers, it was found that a child was unable to learn a new word from his mother if the process was interrupted by mobile phone mother’s use [48]. Today we speak more broadly of “technoference” in reference to this interference of digital tools in human relations at any age, as previously noted in regard to background television, e-books and animated tablet games [49–51]. In a recent article, Radesky consequently proposed “putting limits to the screens ... for parents” [52]. Effectively these parental screen-interactions compete deeply with the essential parent-children interactions.

### The role of the screen design in deleterious effects is underestimated

New digital objects, by the presence of the applications they support, have a more “captivating” effect that must also be taken into account. This is in fact inherent to the very genesis of these new systems; we know today that many applications and platforms originally intended for adults have been conceived, designed, and are frequently improved with the intention of catching the attention of the user. Such intention is often termed “persuasive design” [53]. Access to the screen is increasingly intuitive and fast; game applications combine a permanent adaptation of the game level to that of the player with reward systems, two elements that are known to be essential in ensuring activities like gambling are addictive. These “attention capturing” features are not excluded from digital content intended for children. At the end of watching a video on the most used “On-demand video streaming services “for children, a new video starts automatically, and it is known that this function increases the number of videos watched by a user. Clearly, these different dark patterns grabbing parents’ and children’s attention strengthen the screen-interactions, worsen the deleterious effects on family interactions.

The English foundation “5rights” would like to reconsider the “rights of the child” in the digital age by banning these addictive elements on applications, sites and social networks for children [54].

### New recommendations about technoference, interactivity and design are needed

Analogous with previous studies carried out on television, studies in recent years on the effects of new mobile and interactive displays on child development portray negative findings. Perhaps in the absence of adequate information, parents continue to use these new screens without any real positive objective for the child, but rather to get free time, to calm their child, or for their child’s immediate enjoyment. These devices disrupt and take away from other essential parts of a child’s time; such as learning time, sleep time, physical activity, and free play time. Even more disturbing, the interactivity of these new devices, which once seemed promising, has complex consequences, including a reduction in parent/child interactions and diminished attention spans. Actually, screen-interactions are becoming more and more pregnant and are done to the detriment of interactions between parents and children.

To conclude, the gravity of these clinical findings confirms the importance of the former recommendations of the various pediatric academies worldwide who, for a long time or more recently, have clearly discouraged screen exposure for very young children [55, 56]. They have still their importance and must be largely shared: no media use in children under 2 years of age, during meals and at least for 1 h before bedtime, etc..

However, in the light of this latest data about new media, further recommendations which include specific precautions about technoference and care about interactivity and design’ issues must be drawn up for parents. Effectively these new problematics are largely unknown by the public and do not appear explicitly in the European or American screen guidelines [55, 57, 58]:

### About technoference

Advise parents in the maternity ward to put their cell phones screen facing down as long as their baby is awake.

Recommend to parents not to let their screen interactions compete with real interactions with their child which are the core of learnings (especially language) and emotional regulations.

Remind parents that when they are on their cell phone, they may be deeply distracted, and that the security of their child cannot be ensured.

### About interactivity and persuasive design

Advise parents to avoid e-books, applications, games or platforms with too high levels of interactivity or which use persuasive technologies to hook children’s attention. At least to be aware about dark patterns, to limit them as much as possible (eg: disable autoplay for online videos).

## Data Availability

None.

## Abbreviations

TV: television
CSM: Common Sense Media
